# 125 years of the Wertheim operation. What next?

**DOI:** 10.25122/jml-2022-0082

**Published:** 2023-03

**Authors:** Emmanuel Drouin, Jean-Marc Classe, Patrick Hautecoeur

**Affiliations:** 1 Neurology Service, Lille Catholic Institute Hospital Group, Lomme cedex, France; 2 Ouest Cancer Institute, Saint Herblain, France; 3 Department of Cancerology, Medical University, Nantes, France

**Keywords:** Wertheim surgery, history of medicine, oncology

## Abstract

The Austrian gynecologist Ernst Wertheim (1864-1920) was a pioneer in the surgical treatment of cancer. The principle of Wertheim’s hysterectomy was to remove the uterus and the cervix with appropriate parametrium and tissues surrounding the upper vagina and pelvic lymph nodes. However, in the early 2000s, a meta-analysis of randomized trials revealed that radiotherapy and concomitant chemotherapy without surgical removal of the uterus were more effective in the historical treatment of advanced cervical cancer. This finding challenged the use of radical abdominal hysterectomy (RAH) in such cases and demonstrated the superiority of radiotherapy and chemotherapy in terms of overall survival.

## INTRODUCTION

Vaginal hysterectomy was first mentioned in Gynaecology, written by Soranus of Ephesus (1st/2nd century AD), a Greek physician [1]. Later, Avenzoar (1091-1162) described a total extirpation of the uterus [2], and in 1507, Giacomo Berengario da Carpi (1460-1530) performed a partial hysterectomy in a case of prolapse [3]. Conrad Langenbeck (1776-1851) achieved the first successful planned vaginal hysterectomy in 1813 for endometrial carcinoma, while Charles Clay (1801-1893) was among the earliest successful practitioners of abdominal hysterectomy in Europe [4]. Wilhelm Alexander Freund (1833-1917) performed the first known abdominal extirpation of a cancerous uterus in 1878, and he subsequently standardized the technique for total abdominal hysterectomy. The primitive form of radical hysterectomy was first described in 1895 by John Clark and Emil Ries (Chicago) after discovering cervical cancer in the tissues and lymph nodes beyond the excision limits of the standard hysterectomy [5].

## ERNST WERTHEIM

**Figure 1:**
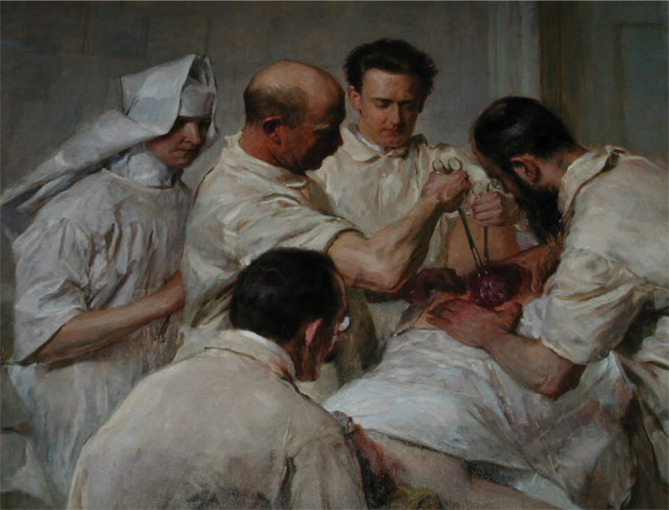
Ernst Wertheim. Paint by von John Quincy Adams. 1906.

Ernst Wertheim (1864-1920), an Austrian gynecologist, was a pioneer in the history of the surgical treatment of cancer. On November 16, 1898, he performed the first radical abdominal hysterectomy (RAH) for cervical cancer (Figure 1) [6]. The principle of Wertheim’s hysterectomy was to remove the uterus and the cervix with appropriate parametrium and tissues surrounding the upper vagina and pelvic lymph nodes. The name of this gynecologist remains eponymously associated with RAH. The "Wertheim operation" became a commonplace, albeit risky procedure for cervical cancer. The early 20th century saw a dispute between Wertheim and Friedrich Schauta (1849-1919) over the best surgical approach for cervical cancer, namely the vaginal or abdominal approach. This disagreement was known as the "Wertheim/Schauta" controversy. In 1901, Schauta described the radical vaginal hysterectomy and reported a lower operative mortality rate than the abdominal approach.

Radical abdominal hysterectomy (RAH) as a carcinoma treatment was ultimately abandoned due to the emergence of radiotherapy [7,8]. However, it was later modified and repopularized by Joe Vincent Meigs (1892-1963) in the 1950s, adding more extensive pelvic lymphadenectomy. The Wertheim-Meigs surgical approach has been used by gynecologic oncologists until today [9]. Advances in anesthesia, blood transfusion, antibiotics, and surgical techniques led to hysterectomy becoming the second most common operation in women, with different types of radical hysterectomy [10].

Managing carcinoma of the uterine cervix presents a rare example of a surgical technique that has been promoted, discarded, rehabilitated, and ultimately abandoned once again. Recent randomized trials have shown that for early-stage cervical cancer, mini-invasive surgery (laparoscopy or robotic) to remove the uterus is detrimental compared with laparotomy, resulting in higher mortality rates and more loco-regional relapses [11]. One of the main hypotheses behind this outcome is that during mini-invasive surgery, the surgical tools may come into contact with and rupture the tumor, which may then spread. Herman Boerhaave (1668-1738), in his surgical aphorism 504, advised that: "cancer must not be touched unless it can be completely eradicated". RAH, performed during a laparotomy, enabled complete tumor removal with minimal contact. The concept of complete tumor removal remains of the main principles of surgical oncology, first described by Herman Boerhaave, who stated: "*If we cannot entirely eradicate the cancer with its roots and its seeds, it becomes irritated, enters within, produces other cancers and increases those which have formed*" [12].

In the early 2000s, a meta-analysis of randomized trials demonstrated the superiority of radiotherapy and concomitant chemotherapy without surgical removal of the uterus over the historical treatment of advanced cervical cancer with external radiotherapy and RAH in terms of overall survival [13]. This finding is a scientific demonstration, 2400 years later, of Hippocrates’ hypothesis in his Aphorism 38: "*We must waive the operation to extirpate the cancer, a useless and even harmful operation, and we must limit ourselves to the use of remedies*" [14]. Current international guidelines recommend avoiding radical hysterectomy in patients treated for advanced cervical cancer, and instead, referral for definitive chemoradiotherapy is advised [15]. This is a notable example of the evolution of surgical oncology, where the initial focus was solely on the efficiency of tumor removal as the primary treatment. However, as consideration of the risks and development of complementary treatments became more important, the balance between treatment efficiency and impact on patient quality of life led to surgical de-escalation.

Finally, in the preamble to his conference on the 7th March 1896 at the Nantes School of Medicine, Dr. Stéphane Leduc (1853-1939), a doctor and physicist from Nantes, and friend of Marie Curie, specified: "*It is necessary to subject one’s mind to a veritable metamorphosis: it is necessary to abandon the opinion that the world is such as we see around us, such as we reveal to our senses. In reality, there exists an infinity of things and phenomena that we overlook from all angles. Science reveals to us the existence of beings and phenomena that we have let our senses ignore*" [16].

The words of Dr. Stéphane Leduc, spoken over a century ago, still hold relevance today to innovate in cancerology.
